# High-brightness Cs focused ion beam from a cold-atomic-beam ion source

**DOI:** 10.1088/2399-1984/aa6a48

**Published:** 2017-05-02

**Authors:** AV Steele, A Schwarzkopf, JJ McClelland, B Knuffman

**Affiliations:** 1 zeroKNanotech, Gaithersburg, MD 20879, United States of America; 2 Center for Nanoscale Science and Technology, National Institute of Standards and Technology, Gaithersburg, MD 20899, United States of America

**Keywords:** focused ion beam, ion source, laser cooling, nanofabrication

## Abstract

We present measurements of focal spot size and brightness in a focused ion beam system utilizing a laser-cooled atomic beam source of Cs ions. Spot sizes as small as (2.1 ± 0.2) nm (one standard deviation) and reduced brightness values as high as (2.4 ± 0.1) × 10^7^ A m^−2^ Sr^−1^ eV^−1^ are observed with a 10 keV beam. This measured brightness is over 24 times higher than the highest brightness observed in a Ga liquid metal ion source. The behavior of brightness as a function of beam current and the dependence of effective source temperature on ionization energy are examined. The performance is seen to be consistent with earlier predictions. Demonstration of this source with very high brightness, producing a heavy ionic species such as Cs^+^, promises to allow significant improvements in resolution and throughput for such applications as next-generation circuit edit and nanoscale secondary ion mass spectrometry.

## 1. Introduction

Ion beams focused to nanoscale dimensions have become an essential tool for nanotechnology, spanning a wide variety of disciplines ranging from three-dimensional imaging and analysis of samples in biology, geology and materials science, to circuit diagnosis and repair in state-of-the-art semiconductor manufacturing. Over the course of several decades, the gallium liquid metal ion source (LMIS) has become the most common approach to producing these beams, with its simple construction, high brightness and stable output. For many applications, the Ga LMIS continues to be the source of choice, with several commercial versions available, and a robust literature of source characterizations and application demonstrations [1–9].

Recently, an increased demand for higher performance focused ion beams (FIBs) has emerged. Growing requirements for higher resolution, more beam current density, and better control over sputtering and damage have led the research community to develop a number of alternatives to the Ga LMIS. For example the He gas field ion source (GFIS) [10, 11], and more recently the Ne GFIS [12], have significantly higher brightness than the LMIS and can produce correspondingly smaller spot sizes. Inductively coupled plasma sources have also become available [13]. While these plasma sources do not have a brightness as high as the LMIS or the GFIS, they have the advantages of a higher current and access to a high-sputter-yield, low-contamination, heavy ion species such as Xe. The extension to species other than Ga is an important one, as it opens possibilities for not only optimizing sputter yield and controlling contamination, but also for selective nanoscale implantation of specific species [14]. To this end, extension of the LMIS to alloys has recently seen development, expanding the possibility to as many as 46 different ionic species, with the incorporation of a mass filter in the source [15].

Sources based on ionization of laser cooled atoms have recently attracted attention in the search for higher brightness, access to new ionic species, and overall better performance [16]. With this type of source, neutral atoms are cooled using momentum transfer from near-resonant laser light [17] to temperatures in the microkelvin range, and then ionized via a focused laser beam to create a very high brightness ion beam. Advantages of this approach include a brightness that does not rely on producing ions from a potentially unstable very sharp tip, an inherently narrow energy spread, and well-developed technology for laser cooling over 27 ionic species, many of which are not easily addressed with alloy LMIS, GFIS, or plasma sources. It is also worth noting that while extraordinarily cold temperatures are involved in this type of source, laser cooling allows this to be accomplished without the need for cryogens of any sort.

Two types of cold atom ion source have been successfully demonstrated based on this principle. In a magneto-optical trap ion source (MOTIS) [18, 19], atoms are cooled and collected in a three dimensional trap, consisting of a quadrupole magnetic field and three pairs of counter-propagating laser beams incident from three orthogonal directions, before being ionized by one or more additional lasers. This type of source has been realized with Cr [20, 21], Rb [19, 22–24], and Li [25, 26]. Alternatively, an atomic beam of neutral atoms can be cooled to microkelvin temperatures in only the two transverse dimensions before entering an ionization region [27]. This approach overcomes a limitation on the current produced in a MOTIS arising from the slow transport of cold atoms into the ionization region [28], since a constant flux of atoms is available from the beam. Successful demonstrations of this type of source producing Cs ions have recently appeared [29–31], showing great promise.

In this paper we present measurements on a Cs cold atomic beam ion source in a so-called LoTIS configuration [32], demonstrating a brightness well over 2 × 10^7^ A m^−2^ Sr^−1^ eV^−1^—as much as 24 times higher than that seen with the Ga LMIS—and show spot sizes in the single-digit nanometer range. These results represent a breakthrough in heavy ion source development. Up to now, the Ga LMIS, with its maximum brightness [9] of 10^6^ A m^−2^ Sr^−1^ eV^−1^, has remained the most practical choice for high speed, high resolution milling. The introduction of this new type of Cs ion source will enable a higher flux of ions in a smaller spot size with a larger sputtering rate per ion, realizing much improved throughput and resolution.

Although the LoTIS has a number of inherent advantages over a Ga LMIS besides a higher brightness, such as a lower energy spread and potential for very long term stability, the fact that it can be implemented with Cs is particularly advantageous. For example, table 1 shows Monte Carlo calculations [33] of sputter rate, ion depth, and straggle at 30 keV incident energy for several ion species accessible with high brightness sources. As seen in the table, Cs is calculated to have a 31% greater sputter rate, while maintaining 14% smaller penetration depth and 36% smaller straggle, when compared with Ga. The comparison with Ne and He is even more favorable. This comparison shows that better milling performance can be expected when using Cs. In addition, a LoTIS-based Cs source should prove very useful for nanoscale secondary ion mass spectrometry (SIMS) applications, where Cs is the ion of choice for studying electronegative species. Current Cs ion sources used in SIMS have typical brightness [34] in the range of 500 A m^−2^ Sr^−1^ eV^−1^, so the LoTIS could represent a more than 10^4^ fold improvement.

## 2. Experiment

**Figure 1:**
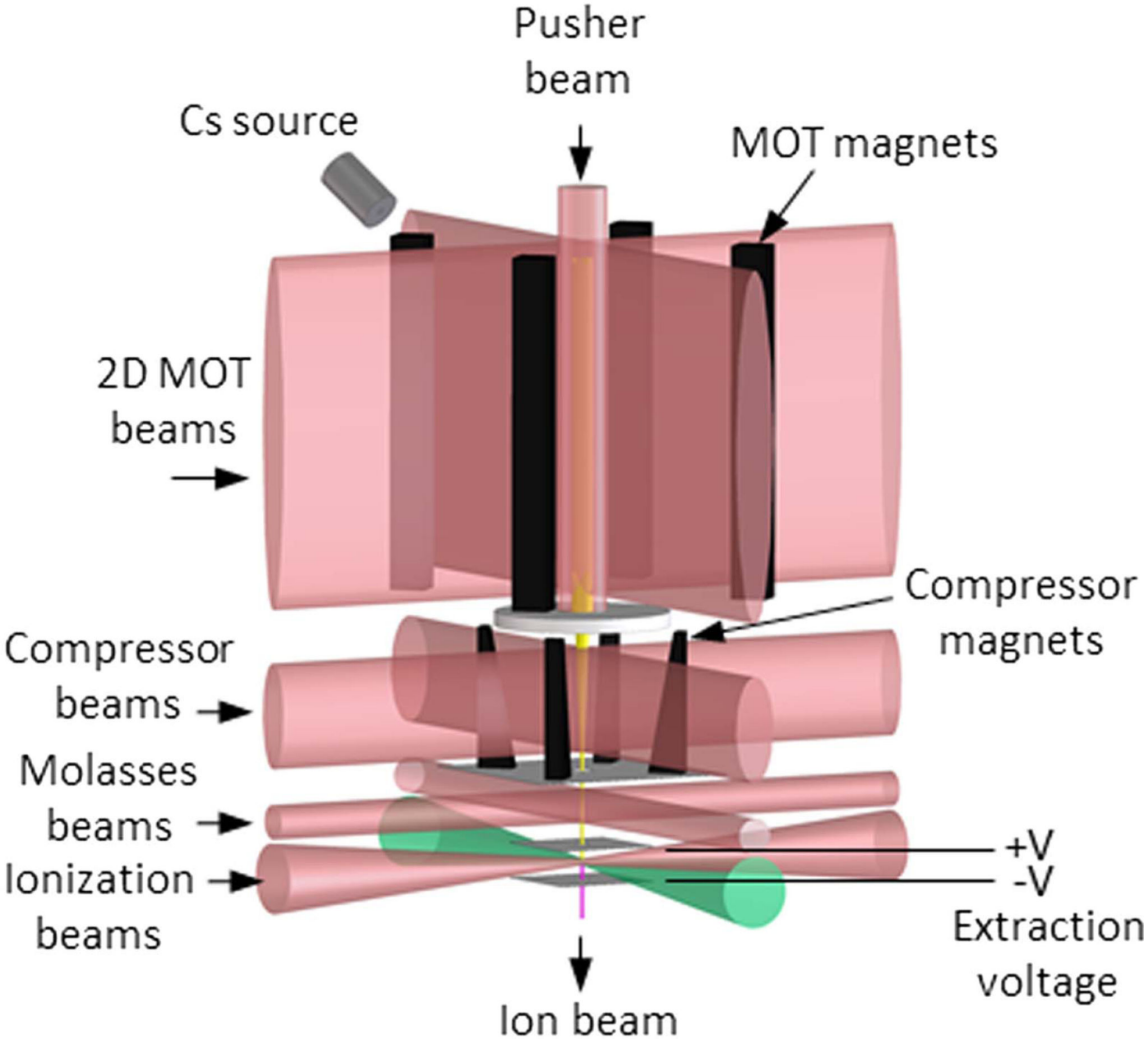
Schematic of LoTIS cold atomic beam ion source. Cs atoms are trapped and cooling in a 2D magneto-optical trap (MOT), pushed into a magneto-optical compressor, further cooled in polarization-gradient optical molasses, then photoionized in a two-step process and extracted with an electric field.

The LoTIS, figure 1, has been described in previous publications [29, 32]. Briefly, Cs vapor from a heated Bi–Cs alloy source enters a room-temperature rectangular glass cell where it is captured and cooled in a two dimensional magneto-optical trap [35]. A ‘pusher’ laser beam tuned to the Cs atomic resonance at 852 nm and oriented along the axis of the trap creates a high-flux, slow atomic beam with mean velocity approximately 10 m s^−1^, which exits through a 1 mm aperture. The beam then enters a magneto-optical compressor [36], which is essentially another two-dimensional magneto-optical trap with increasing magnetic field gradients along the beam axis. The compressor reduces the beam diameter to a few tens of micrometers, resulting in a peak atomic flux of nearly 10^18^ m^−2^ s^−1^. The beam then enters a magnetically shielded region where it is further cooled in two dimensions using polarization gradient optical molasses [37] to a temperature of ^4^ (10 ± 3) *μ*K.

Ionization occurs in the next section, where two conducting plates with apertures create an extraction electric field. A pair of focused, crossed ionization laser beams, one tuned to the Cs resonance near 852 nm, and the other tuned near 508 nm, ionizes the atoms in a small volume created by the overlap of the foci of these two beams. The 508 nm laser frequency is chosen to promote 6P_3/2_ Cs atoms excited by the 852 nm laser beam to an energy level between the field-free ionization threshold and the classical ionization saddle point set by the extraction field [38]. The exact frequency is chosen based on balancing the relative need for low ion temperature or high ion current. The focal spot sizes of the 852 nm and 508 nm ionization laser beams are chosen in a range between 8 and 160 *μ*m (1 *e*^2^ diameter), depending on the desired current and resolution, with the larger diameters yielding higher currents at somewhat lower brightness, due largely to Coulomb effects. After exiting the ionization region at a beam energy of up to 1 keV, the ion beam enters a set of electrostatic lenses where it is accelerated to the desired energy and formed into a beam with the desired diameter and divergence.

The entire LoTIS is mounted in place of a Ga LMIS on a commercial high resolution FIB column, with the usual condenser lens, stigmators, deflectors, and objective lens. In our case the condenser lens is not used because sufficient control over the beam divergence is provided by the LoTIS acceleration optics. There is no need to use a beam limiting aperture in this system; because there is no minimum emission associated with the source, controlling the ion source current is instead matter of choosing the power and geometry of the ionizing laser beams.

The energy spread of an ion source is an important consideration because it can lead to limitations of the spot size due to chromatic aberration. Unlike the LMIS, which has an energy spread of 4–5 eV arising from the emission characteristics of the Taylor cone and Coulomb interactions when the typical current of 2 *μ*A is extracted, the LoTIS has a very small inherent spread, dominated by the extraction potential gradient across the ionization laser beams’ extent along the direction of the field. Previous measurements [32] have shown this spread to be in the range of 0.45 eV with an extraction field of 100 kVm^−1^. This small energy spread contributes to the enhanced performance of the source, since it makes it possible to use a larger convergence angle in the focused beam without introducing excessive chromatic aberration.

## 3. Results

Our initial prototype source was designed and constructed to operate at 10 keV ion beam energy. While resolution can always be improved by increasing ion beam energy, the initial purpose of the prototype is to carry out measurements of spot sizes and convergence angles with the aim of characterizing the source’s brightness. A 10 keV beam is entirely adequate for this purpose.

**Figure 2:**
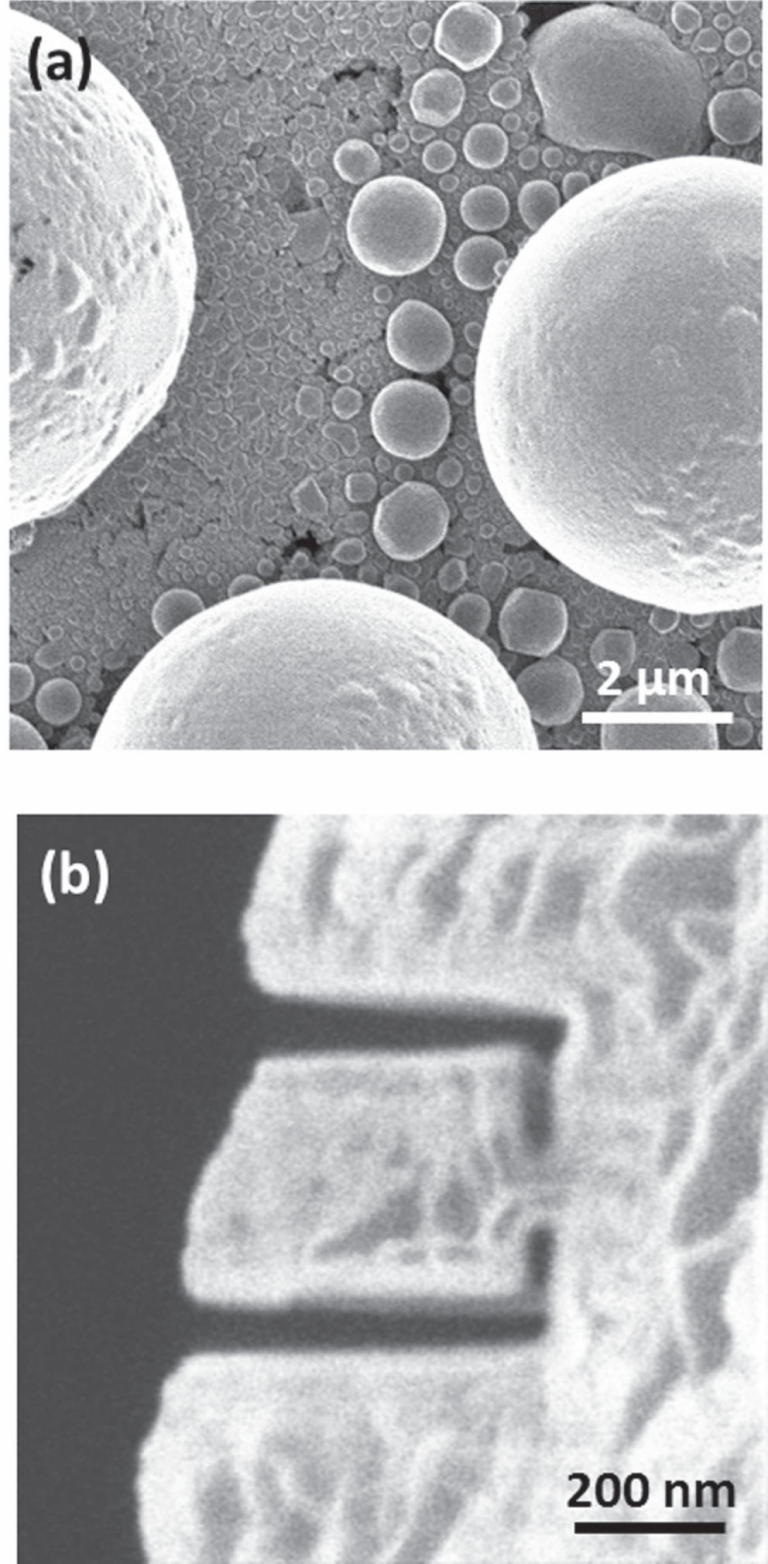
(a) Secondary electron image of a standard tin ball resolution target acquired using a focused 10 keV, 1 pACs^+^ ion beam from the LoTIS. (b) Secondary electron image of a pattern milled in the edge of a Cu grid using a similar Cs^+^ ion beam. Milling time for this pattern was approximately 120 s.

Figure 2 contains images exemplifying the qualitative performance of the source. In figure 2(a) we show a scanning ion micrograph of a standard tin ball microscopy resolution sample, acquired by collecting secondary electrons while a scanning a 10 keV, 1 pA ion beam. This image, acquired in a single scan over 17 s, illustrates the level of beam stability and resolution that can be obtained with a LoTIS. Figure 2(b) shows a scanning ion micrograph of a pattern milled by a similar ion beam, demonstrating the milling capability of a 10 keV Cs^+^ beam. We show this just as an example, although optimum resolution and milling rate may well be achieved at a higher beam energy.

### 3.1. Spot size measurements

**Figure 3:**
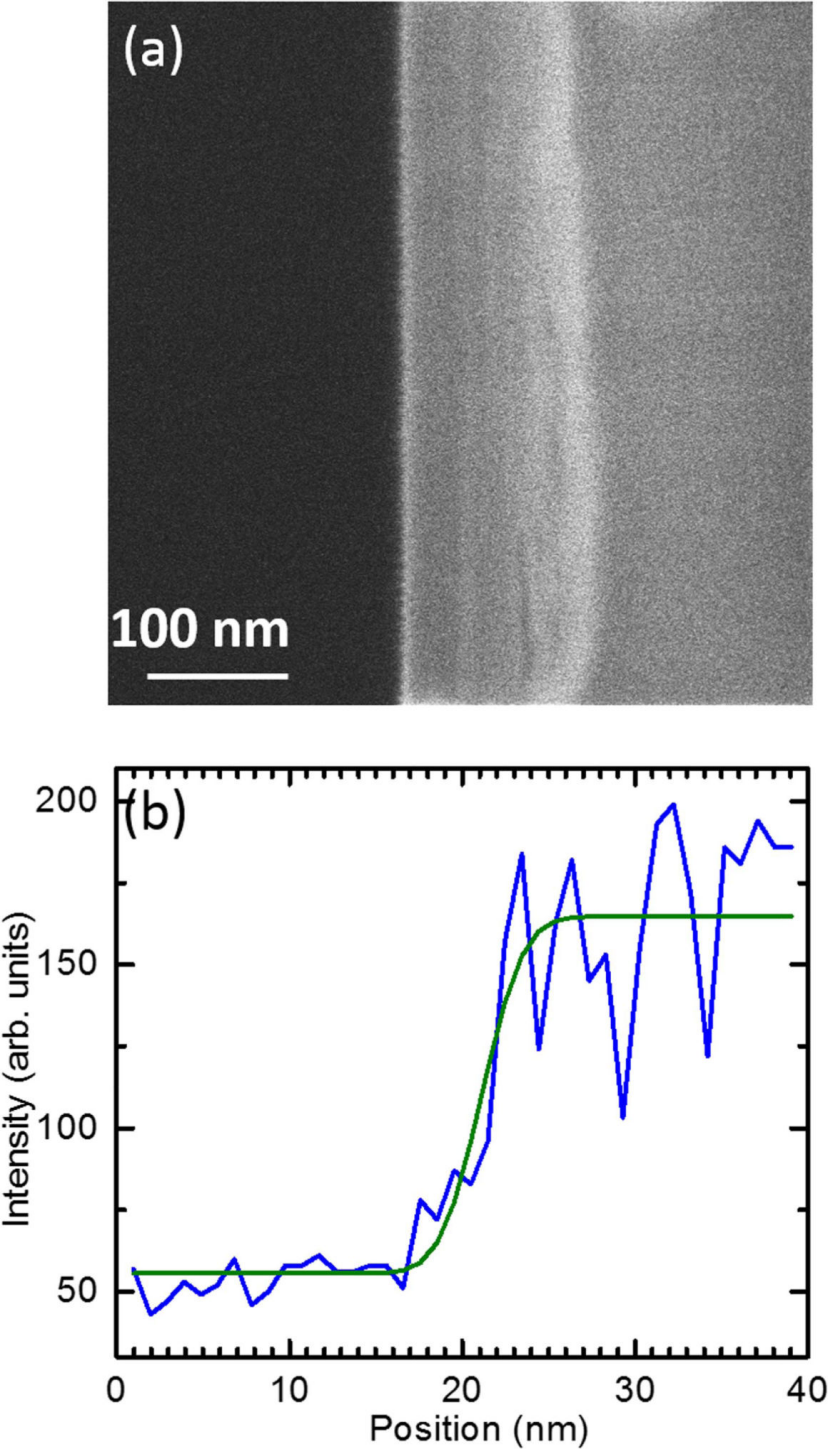
Measurement of spot size by scanning ion beam across edge of cleaved Si wafer. (a) Secondary electron image of wafer edge; (b) single line scan (blue) and fit to error function (green).

After optimizing the ion optics for best resolution, spot sizes were measured by scanning the ion beam across the edge of a cleaved Si wafer and collecting secondary electron emission (figure 3). For each horizontal line scan in the image, a fit was made to an error function plus background:

(1) $$S(x)=A+\frac{B-A}{2}[1-\text{erf}(\frac{x-x_{0}}{\sqrt{2}\sigma_{x}})],$$

where *S* (*x*) is the signal as a function of pixel position *x*, and the dark level *A*, the bright level *B*, the centroid *x*_0_, and the standard deviation *s_x_* are free parameters. Allowing the centroid to be a free parameter for each scan reduced the effects of sample vibrations and/or beam position instabilities, which were present at the level of approximately 5–10 nm, but on a time scale much slower than the transit time of the scan across the edge. The resulting beam widths were averaged across the image. After exploring the parameter space of acceleration optics, focus and stigmation settings, the smallest observed focal spot for a 10 keV, 1.2 pA beam was found to have a standard deviation of (2.1 ± 0.2) nm (1.6 nm 35–65 width, or 2.8 nm 25–75 width)^5^. The uncertainty in this value contains statistical variation from line scan to line scan, as well as systematic components arising from possible beam focus errors, and is intended to be interpreted as one standard deviation.

While scanning the beam across an edge is a common method for FIB spot characterization, it can sometimes be misleading if milling of the edge occurs during the measurement [39]. Milling-induced systematic effects on the spot size result were investigated by reversing the direction of the beam scan over the silicon edge; the spot size measurement was not found to be dependent on this choice of direction. It is believed that in our case milling was minimal due to the relatively low 10 keV beam energy, the low beam currents (≈1 pA) used for high resolution operation, and the use of the fastest scan speed that could be used while maintaining good signal-to-noise-ratios. Any residual effects were minimized by fitting the edge profile line by line.

### 3.2. Brightness measurements

The reduced, or scaled, brightness of an ion beam is independent of beam energy and does not depend on the presence of apertures in the beam, and thus is a good figure of merit for describing the performance of a source. Given a source’s reduced brightness and energy spread, it is possible to predict the expected spot size for any final beam energy, focal length and convergence angle in a given focusing scenario, provided the chromatic and spherical aberration coefficients of the lens are known [40]. There are several definitions for a beam’s reduced brightness in the literature, with coefficients depending, for example, on whether the beam has a uniform, Gaussian, or other distribution. For present purposes we consider the peak reduced brightness at the center of a cylindrical beam with Gaussian distributions in both the transverse spatial and the angular coordinates. We note this is an appropriate description for a LoTIS, since the ion beam is generated by laser beams with nearly Gaussian distributions and the beam is not defined by any apertures. In this case, we write

(2) $$B=\frac{1}{4\pi^{2}\sigma_{x}\sigma_{y}\sigma_{\theta_{x}}\sigma_{\theta_{y}}U},$$

where *I* is the beam current, *σ_x_* and *σ_y_* are the standard deviation in the transverse directions, *σ_θ_x__* and *σ_θ_y__* are the standard deviation in convergence angles, and *U* is the beam energy [16].

We measure the brightness of the LoTIS as follows. Using the objective lens of the FIB column, we create a focal spot which we measure in the manner described above along two orthogonal axes. We then obtain the convergence angle by turning off the objective lens and scanning the beam across the cleaved Si edge again along those same axes. The spatial distribution of the unfocused ion beam at the sample is a good measure of the distribution at the principal plane of the objective because the ratio of the focal distance to the full length of the column is small (≈0.05), and the divergence of the beam is also small (<3 *μ*rad). The standard deviation of the convergence angle is then derived from the standard deviation of the spatial distribution at the principal plane *σ_L_* and the focal length of the lens *f* (in our case 30 mm) via *σ_θ_* = *σ_L_/f*. Combining this with the measured beam current, energy and standard deviation of the focal spot *σ_x_* in equation (2) yields the peak reduced brightness.

**Figure 4:**
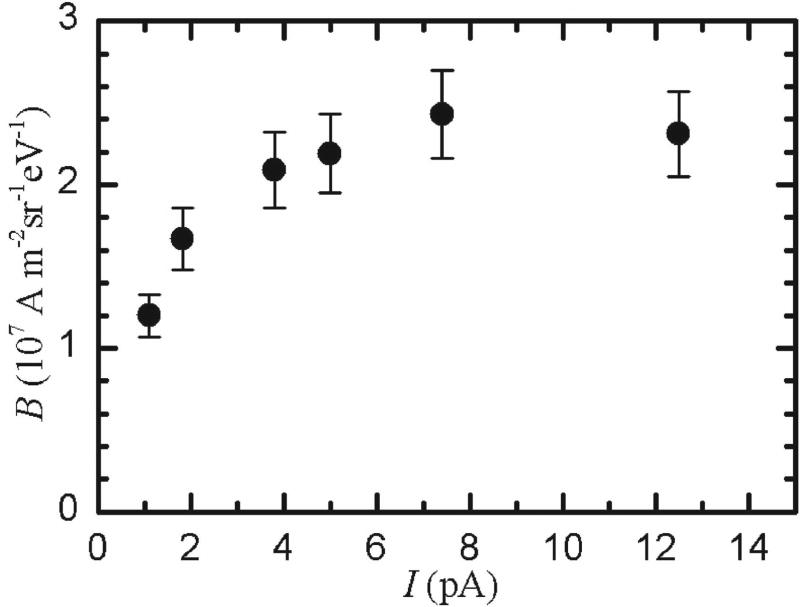
Measurements of peak reduced brightness, *B* as a function of beam current, *I*, varied by adjusting ionization laser power. Error bars indicate one standard deviation uncertainty, derived by combining uncertainties in measurements of current and spot sizes.

For the purpose of brightness measurement, the system was operated in a regime where the beam exits the accelerator essentially collimated, with a relatively small diameter in the objective lens (≈2.4 *μ*m). This regime was chosen so that the focal spot size would be dominated by the beam brightness, and the contributions from aberrations in the column would be negligible. With this beam configuration—which is different from the configuration chosen for measuring the smallest focal spot discussed above—the focal spot is typically larger than 5 nm. The chromatic aberration contribution is estimated to be less than 0.5 nm, and the spherical aberration contribution is three orders of magnitude smaller. Several measurements were also performed over a range of opening angles that included the ones used in figure 4; these brightness values did not vary appreciably, as they would have if aberrations were significant. This larger spot size is additionally advantageous because it minimizes the possible impact of sample interactions or environmental perturbations on the results.

The largest reduced peak brightness observed was (2.4 ± 0.1) × 10^7^ A m^−2^ Sr^−1^ eV^−1^ with a 7.4 pA beam operating at 10 keV. Figure 4 shows brightness measurements as a function of beam current, where the current was varied by changing the ionization laser powers. For these measurements, the ionization laser spot sizes and accelerator voltages were held fixed for all measurements. The brightness falls off at below 2 pA because the ionization efficiency is smaller at lower ionization laser intensity. It is important to note that the lower brightness at 1.0 pA does not represent a fundamental limitation of the system. Higher brightness could in principle be obtained at lower currents by focusing the ionization laser more tightly [29].

Given the maximum brightness measured of 2.4 × 10^7^ A m^−2^ Sr^−1^ eV^−1^, even smaller focal spot sizes should in principle be achievable than the 2.1 nm spot size demonstrated to date. We believe that platform and environmental difficulties account for this discrepancy. In addition, it is possible that aberrations due to ions optical misalignment or fabrication tolerances are degrading focusing performance for very small probe sizes. Achieving the nearly 1 nm spot sizes that the above brightness and energy spread will likely require a FIB platform and source accelerator optics with more stringent design specifications.

### 3.3. Source temperature

A measurement of the effective transverse ion temperature is of interest to help clarify whether the underlying cold atom temperature is dominant, or whether other effects such as Coulomb interactions cause additional heating. It is possible to extract the effective transverse temperature of the ions leaving the source, *T*, by equating the emittance at the source, $\sigma_{0}\sqrt{k_{B}T/2}$, to the emittance at the focus, $\sigma_{x}\sigma_{\alpha }\sqrt{U}$, (*k*_B_ is Boltzmann’s constant) [25]. Defining the effective focal length *f* of the objective as *f* ≡ *σ*_0_/*σ_α_*, we can write

(3) $$T=2\frac{U}{k_{B}f^{2}}\sigma_{x}^{2}.$$

We used equation (3) to obtain measurements of *T* by focusing the ion beam onto the cleaved Si edge using the accelerating optics near the ion source. Ray tracing simulations were used to determine the effective focal length *f* of this accelerating lens configuration, and the rise distance of the secondary electron signal was used, as described above, to characterize the standard deviation of the current distribution at the focal spot *σ_x_*.

**Figure 5:**
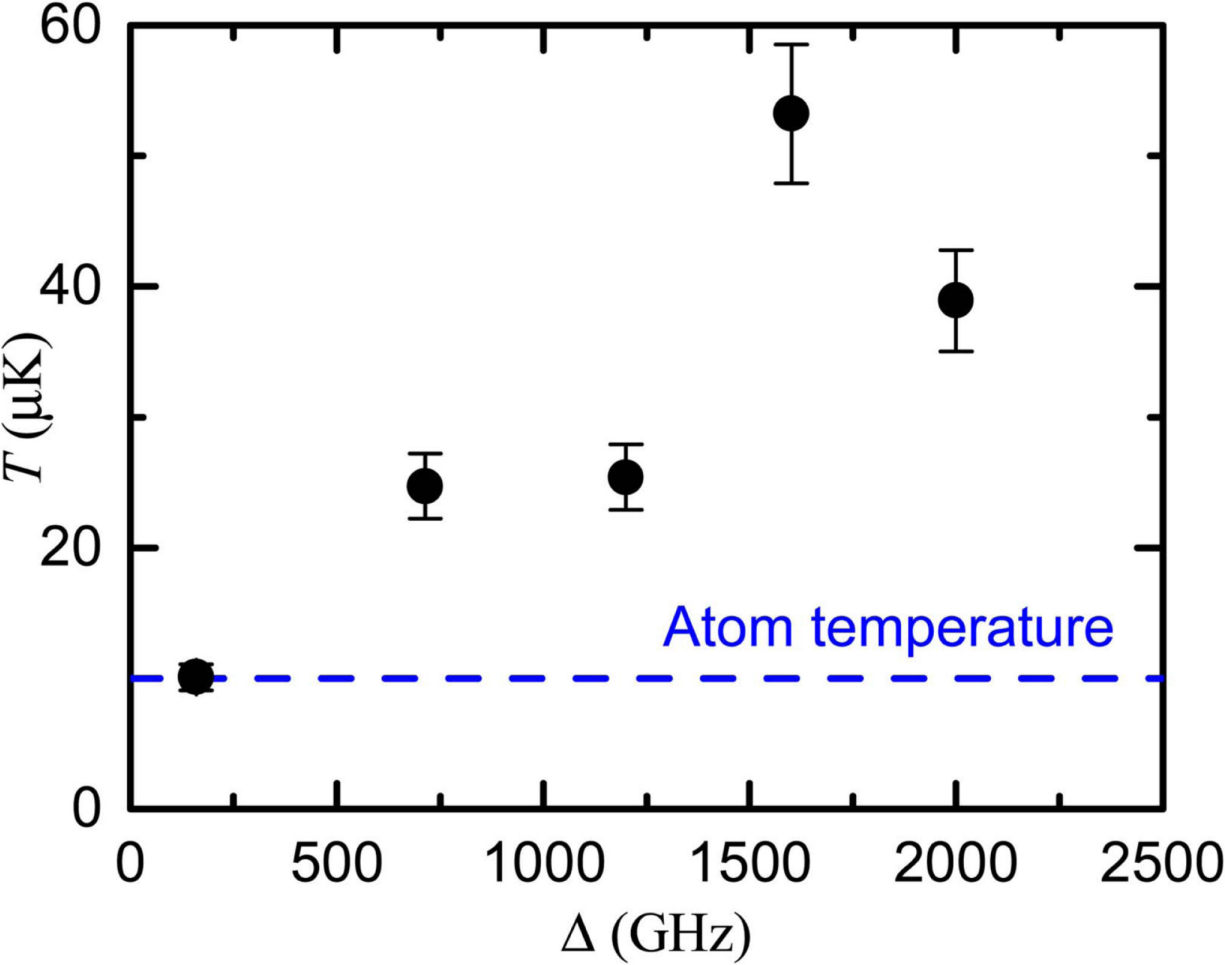
Ion temperature *T*, as a function of ionization laser detuning Δ above the classical field ionization threshold. The atom temperature is shown with a dashed blue line. Error bars indicate one standard deviation uncertainty, derived by combining uncertainties in measurements of current and spot sizes.

Figure 5 shows the derived ion temperature of a 10 keV, 1 pA beam as a function of ionization photon energy, measured in gigahertz detuning above the classical field ionization threshold. Also shown in the figure is a line indicating the transverse temperature of the neutral atoms as they emerge from the polarization gradient optical molasses, measured by turning off the ionization lasers and observing the beam width using laser induced fluorescence after expansion for a distance of 140 mm. Close to threshold, the measured ion temperature is seen to be consistent with the atom temperature of 10 *μ*K. At higher photon energies, the ion temperature increases, presumably as excess photon energy begins to add recoil energy to the ions.

## 4. Summary and conclusion

In this paper we have presented measurements on a laser-cooled atomic beam LoTIS Cs ion source, demonstrating a peak reduced brightness as high as (2.4 ± 0.1) × 10^7^ A m^−2^ Sr^−1^ eV^−1^. We have also measured spot sizes as small as (2.1 ± 0.2) nm using a 10 keV beam, and shown example images and milling patterns. The brightness measurements confirm earlier predictions of the performance of this type of source [29, 32] and demonstrate its potential for producing a high performance FIB. The brightness attained by this source is significantly higher than LMIS or plasma sources, suggesting that smaller spot sizes and higher milling rates can be attained.

While the results presented here demonstrate improved performance over other sources, it should be noted that the system discussed here is still not fully optimized. With further optimization, it is reasonable to expect that even smaller spot sizes will be possible. The maximum brightness value observed in this work is entirely consistent with creating a sub-nanometer focal spot with a 30 keV, 1 pA beam [32].

Work is ongoing on optimizing this source, with the next steps being demonstration of even smaller focal spot sizes at higher beam energies and also exploring the utility of the source for traditional FIB applications such as circuit edit, transmission electron microscope sample preparation, and general nanofabrication. As improvements to the source continue, its high resolution, along with its ability to produce a wide range of currents from picoamperes to nanoamperes, promise to open an even broader array of applications in present and next-generation nanotechnology.

## Figures and Tables

**Table 1:** Monte Carlo calculations of sputter rate, implantation depth and straggle for 30 keV ions of four species typically used in high resolution focused ion beams. Because of its heavy mass, Cs has a higher sputter rate and smaller penetration depth and straggle than the other species. 10 000 ion impacts were calculated in each case and statistical variations were less than the precision of the numbers in the table.

Species	Mass (u)	Sputter rate (atom/ion)	Depth (nm)	Straggle (nm)
He	4	0.02	211	62
Ne	20	0.92	67	29
Ga	70	2.3	28	10
Cs	133	2.9	24	6.6

## Footnotes

4 All uncertainties in this paper are intended to be interpreted as one-standard-deviation, combined standard uncertainty.

5 The 35–65 width is defined as the distance over which the edge profile rises from 35% to 65% of the maximum value. The 25–75 width is similarly defined for a rise of 25%–75%.
